# Adipogenic Transdifferentiation and Regulatory Factors Promote the Progression and the Immunotherapy Response of Renal Cell Carcinoma: Insights From Integrative Analysis

**DOI:** 10.3389/fonc.2022.781932

**Published:** 2022-03-09

**Authors:** Shuai Wang, Xiyi Wei, Chengjian Ji, Yichun Wang, Xi Zhang, Rong Cong, Ninghong Song

**Affiliations:** ^1^ The State Key Lab of Reproductive Medicine, Department of Urology, The First Affiliated Hospital of Nanjing Medical University, Nanjing, China; ^2^ Department of Urology, The Affiliated Kezhou People's Hospital of Nanjing Medical University, Kezhou, China

**Keywords:** adipogenic transdifferentiation, adipose-related gene, clear cell renal cell carcinoma, prognostic model, GBP2, immunotherapy

## Abstract

**Background:**

Adipogenic transdifferentiation was an important carcinogenic factor in various tumors, while studies on its role in clear cell renal cell carcinoma (ccRCC) were still relatively few. This study aimed to investigate its prognostic value and mechanism of action in ccRCC.

**Methods:**

Gene expression profiles and clinical data of ccRCC patients were obtained from The Cancer Genome Atlas database. Nonnegative matrix factorization was used for clustering. Gene set variation analysis (GSVA) and gene set enrichment analysis (GSEA) were used to analyze the pathways and biological process activities. single-sample GSEA (ssGSEA) was utilized to quantify the relative abundance of each immune cell. Tumor Immune Estimation Resource (TIMER) was used to evaluate the proportion of various immune infiltrating cells across diverse cancer types. Real-Time PCR was performed to examine the gene expression. R software was utilized to analyze the expression and prognostic role of genes in ccRCC.

**Results:**

A total of 49 adipose-related genes (ARGs) were screened for differential expression between normal and ccRCC tissues. Based on differentially expressed ARGs, patients with ccRCC were divided into two adipose subtypes with different clinical, molecular, and pathway characteristics. Patients in cluster A exhibited more advanced pathological stages, higher expressions of RARRES2 and immune checkpoint genes, higher immune infiltration scores, and less nutrient metabolism pathways. Adipose differentiation index (ADI) was constructed according to the above ARGs and survival data, and its robustness and accuracy was validated in different cohorts. In addition, it was found that the expression of ARGs was associated with immune cell infiltration and immune checkpoint in ccRCC, among which GBP2 was thought to be the most relevant gene to the tumor immune microenvironment and play a potential role in carcinogenesis and invasion of tumor cells.

**Conclusion:**

Our analysis revealed the consistency of higher adipogenic transdifferentiation of tumor cells with worse clinical outcomes in ccRCC. The 16-mRNA signature could predict the prognosis of ccRCC patients with high accuracy. ARGs such as GBP2 might shed light on the development of novel biomarkers and immunotherapies of ccRCC.

## Introduction

Renal cell carcinoma (RCC) influences more than 400,000 individuals per year, accounting for approximately 2.2% of all cancer diagnoses and 1.8% of all cancer deaths worldwide (1). In 2021, approximately 76,000 cancer cases and 13,700 mortalities were estimated to occur in the United States (2). 16% of RCC patients would present with or develop distant metastases and have a 5-year survival rate of 11.6% according to the Surveillance, Epidemiology, and End Results (SEER) database (3).

RCC is categorized generally into two major groups: clear cell renal cell carcinoma (ccRCC) and non-clear cell renal cell carcinoma (non-ccRCC), among which ccRCC makes up about 70% of all cancers of the kidney (4). ccRCC is defined by malignant renal cells with a clear cytoplasm historically, derived from the epithelium of the renal tubules. ccRCC cells possessed an mixture of glycogen and lipid droplets in cytoplasm, accounting for their “clear cell” style under HE staining (5). Reports have shown that ccRCC cells were capable of pluripotent differentiation *in vitro* and underwent adipogenic differentiation preferentially *in vivo* in human patients (6). Meanwhile, lipid deposition in cancer cells promotes a selective advantage to cancer cells, especially in ccRCC (7). Considering that the adipogenic process might play a deleterious role in driving tumorigenesis in lipid-laden ccRCC, Tan et al. assessed the expression of adipokines in ccRCC and discovered the adipokine chemerin, which was encoded by the retinoic acid receptor responder 2 (RARRES2) gene (8). The functions of this chemerin were verified as inhibiting fatty acid oxidation, maintaining intracellular fatty acid levels, preventing ferroptosis, and promoting cellular adipogenic transdifferentiation (8, 9). Their research data suggested that obesity and tumor cells would promote the occurrence of ccRCC through the adipokine chemerin.

The purpose of this study was to explore the role of adipogenic transdifferentiation in the occurrence and development of ccRCC and develop a model to predict prognosis and therapeutic responses in ccRCC patients. Based on the clustering of genes encoding proteins secreted from brown and white adipocytes, we defined two adipose subtypes. Each adipose subtype corresponded to different clinical and molecular characteristics. We developed and validated a combined adipose-related gene (ARG) expression-based adipogenic differentiation index (ADI) for predicting the overall survival (OS) of ccRCC, which was worth getting validated prospectively and utilized in further clinical practice. In addition, we found out one key ARG—GBP2, which might be a potential biomarker in the prediction of the survival of ccRCC patients.

## Methods

### Acquisition of Data Resource

Publicly available transcriptome data and somatic mutation information of patients with ccRCC were downloaded from The Cancer Genome Atlas (TCGA) database (https://portal.gdc.cancer.gov/), including 72 normal cases and 539 tumor cases. Meanwhile, corresponding clinical feature information of ccRCC were also acquired (**Table 1**). A list of 151 genes encoding 143 differentially regulated proteins secreted from brown and white adipocytes were collected from the previous research (10). We defined these genes as adipose-related genes (ARGs).

**Table 1:** Clinical characteristics of clear cell renal cell carcinoma patients in TCGA database.

Clinical features	Variables	Total (n = 539)	Percentages (%)
Age (year)	≤65	353	65.49
	>65	186	34.51
Gender	Female	186	34.51
	Male	353	65.49
Histological grade	G1	14	2.60
	G2	235	43.60
	G3	207	38.40
	G4	75	13.91
	Gx	5	0.93
	Unknown	3	0.56
T classification	T1	278	51.58
	T2	71	13.17
	T3	179	33.21
	T4	11	2.04
Lymph nodes	N0	241	44.71
	N1	16	2.97
	NX	282	52.32
Distant metastasis	M0	428	79.41
	M1	78	14.47
	MX	31	5.75
	Unknown	2	0.37
Clinical stage	Stage I	272	50.46
	Stage II	59	10.95
	Stage III	123	22.82
	Stage IV	82	15.21
	Unknown	3	0.56

A dataset NIHMS1611472 in Excel format was acquired from the National Center for Biotechnology Information (NCBI) as a cohort for validating the prognostic potential of our model. This dataset was submitted by Braun et al. and included 1006 patients’ clinical data and 311 tissue samples (11).

### Identification of Differentially Expressed ARGs

In the preprocessing of the raw data, the “*Limma*” package was used to correct the data and handle the repeated data. Wilcoxon test was applied to analyze the ARG expression profile in tumor samples compared with normal tissue controls. Adjusted *P* < 0.05 and |log2 fold changes (FC)| > 1 were used as the criteria for screening differentially expressed ARGs. All the above operations were carried out in R software (version 4.1.0).

### Establishment of Adipose Subtypes

As an unsupervised clustering algorithm, Nonnegative matrix factorization (NMF) was used to cluster the ccRCC samples (12). To select the best number of clustering, “Brunet” method was adopted. The number of iterations was 30. The point before the greatest variation in cophenetic values was considered as the optimal number of adipose subtypes. To assess the reliability of the clustering results, the “Kaplan-Meier” method was used to calculate the overall survival and median survival time for each subtype. The log-rank test with a criteria level of *P* < 0.05 was applied to analyze the difference in survival between different clusters. Clinical features (T classification, lymph nodes, distant metastasis, clinical stage, histological grade, gender, age, and survival time) were also included in the comparison between adipose subtypes.

### Evaluation of Biological Process Activities and Immune Characteristics of Subtypes

To estimate the variation in pathways and biological process activities between different adipose subtypes, we performed gene set variation analysis (GSVA) with the gene sets of “c2.cp.kegg.v7.4.symbols” downloaded from the Molecular Signatures Database (MSigDB). Adjusted *P* value < 0.05 and |log2FC| > 0.1 indicated a significant difference between two groups of data. The heatmap was drawn with “*pheatmap*” R packages. We applied single-sample gene set enrichment analysis (ssGSEA) to quantify the relative abundance of each immune cell in the ccRCC tumor microenvironment. The gene set was obtained from the research of Charoentong et al. (13), which provided a variety of human immune cell subtypes. We also utilized student’s t-test to identify differential expression of immune checkpoint blockade genes between adipose subtypes.

### Construction of the ADI Based on ARGs

Univariate Cox regression analyses were conducted to find out candidate ARGs, of which expressions were related to the OS of ccRCC patients. Then, LASSO regression analysis was used to obtain several genes with the significant prognosis that could be an independent indicator. In the end, 16 ARGs were screened to construct the ADI, and a multivariable Cox proportional hazards model was used to calculate the risk coefficients of ARGs. The establishment of ADI based on a linear combination of the relative expression level of genes multiplied regression coefficients. After that, patients with ccRCC were stratified into the low risk and high risk groups according to the median ADI value as the risk cutoff value. Kaplan-Meier method was utilized to analyze the OS of these two groups, and log-rank test was used to evaluate the difference between low and high groups. To verify the prediction value of the model, we plotted receiver operating characteristic curves (ROCs). Finally, we validated our model on a dataset provided by another research (11).

### Evaluation of Modeling Gene Correlation and Function

To investigate the relationships of expression between the modeling genes and the immune checkpoint genes in ccRCC, correlation analysis was performed using Spearman’s method. And we observed that the expression of GBP2 positively correlated with most immune checkpoint genes. Using data from Tumor Immune Estimation Resource (TIMER, http://timer.comp-genomics.org/), which was an open resource for evaluating the proportion of various immune infiltrating cells across diverse cancer types, we visualized the correlation between the CD8^+^ T cell and the expression of GBP2 with the method of “quanTIseq”. *P* < 0.05 was as the threshold, and the correlation value varied from -1 to 1, the larger the absolute value, the more relevant. Stratified analyses between clinical features and the expression of GBP2 were also investigated with the log-rank test respectively. Gene set enrichment analysis (GSEA) was conducted using GSEA v4.0.3 (http://www.broadinstitute.org/gsea).

### RT-qPCR and External Validation of Expression Level

The proteomics data and clinical features of cancer patients from the Clinical Proteomic Tumor Analysis Consortium (CPTAC, https://proteomics.cancer.gov/programs/cptac) and the Human Protein Atlas database (HPA, https://www.proteinatlas.org/) were utilized to analyze the protein expression between ccRCC and normal tissues. The real time quantitative polymerase chain reaction (RT-qPCR) was performed for comparison of gene expressions in different ccRCC cell lines and tissues. The PCR run conditions for the detection of GBP2 were used as previously described (14). The primers applied for GBP2 were displayed follow: “Forward: CTATCTGCAATTACGCAGCCT; Reverse: TGTTCTGGCTTCTTGGGATGA”.

### Statistical Analysis

R software (R version 4.1.0) was used to perform all statistical analyses and graphics. The associations of clinical features between different clusters or groups were examined by the chi-square test or Fisher’s exact test. Differences between the two groups of samples were compared by the Wilcox test, while continuous variables were compared by Student’s t-test. Kaplan-Meier method was utilized to analyze the survival curve, and the significance of the difference was determined by the log-rank test. Our scores with a *P*-value < 0.05 and Spearman correlation coefficient > 0.3 were considered to be statistically significant if not specifically stated.

## Result

### Identification of the ccRCC Characteristic of Adipogenic Transdifferentiation

**Figure 1:**
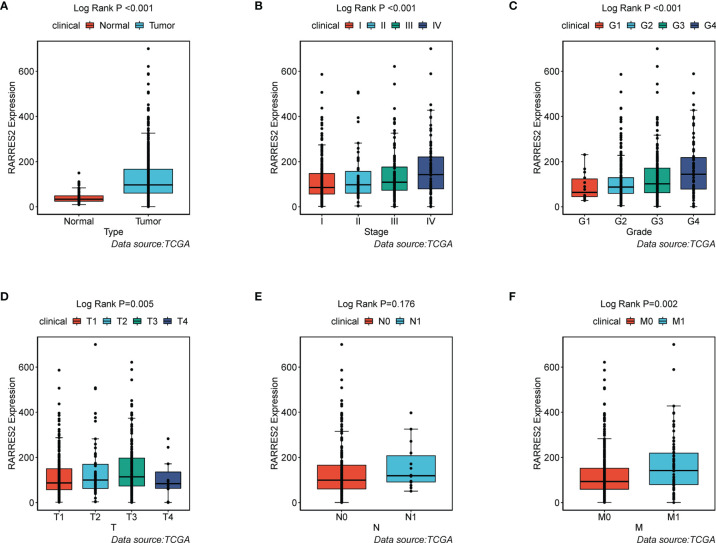
Association of RARRES2 mRNA expression with clinical parameters (data source: TCGA). **(A)** Type (wilcoxon test, *p* < 0.001); **(B)** Clinical stage (K-W test, *p* < 0.001); **(C)** Histological grade (K-W test, *p* = 0.001); **(D)** T classification (K-W test, *p* = 0.005); **(E)** Lymph nodes (wilicoxon test, *p* = 0.176); **(F)** Distant metastasis (wilicoxon test, *p* = 0.002).

To ascertain whether ccRCC was typified by accumulation of neutral lipids and adipogenic transdifferentiation, we firstly investigated the mRNA expression of RARRES2 gene between normal and ccRCC samples. Compared to normal kidney tissues, RARRES2 demonstrated higher expression in ccRCC tissues (**Figure 1A**). We next explored whether RARRES2 expression was associated with clinical features. As was shown in **Figure 1B**, the expression of RARRES2 was correlated positively with the TNM stage of ccRCC patients (*p* < 0.001). There was a significant positive correlation between RARRES2 expression and the histological grade (*p* = 0.001), T classification (*p* = 0.005), distant metastasis (*p* = 0.002), while no significant correlation was found between RARRES2 expression and the lymph nodes (*p* = 0.176) (**Figures 1C–F**). And RARRES2 showed a consistent increase in gene expression and disease severity in all clinical characteristics other than lymph nodes stage or T4 stage.

### Identification of Differentially Expressed ARGs and Molecular Subtypes

**Figure 2:**
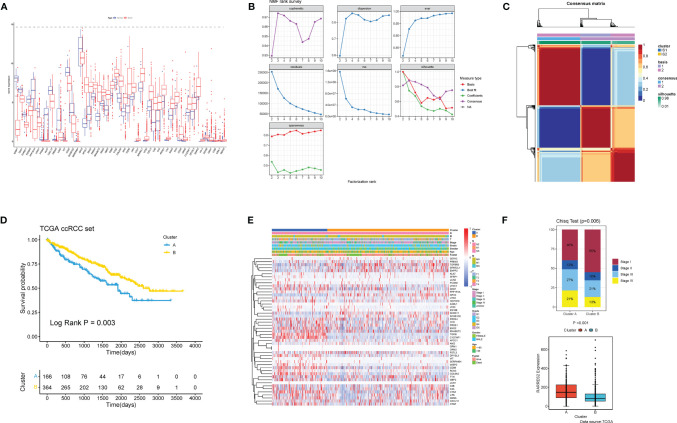
Nonnegative matrix factorization clustering identified two adipose subtypes based on differentially expressed ARGs. **(A)** The expression of 49 differentially expressed ARGs between normal tissues and ccRCC tissues. Tumor, red; Normal, blue. The upper and lower ends of the boxes represented interquartile range of values. The lines in the boxes represented median value. Adjusted *p* < 0.05 and |log2 fold changes (FC)| > 1 were used as the criteria for screening differentially expressed ARGs. The asterisks represented the statistical p value (^*^ *p* < 0.05, ^**^ *p* < 0.01, ^***^ *p* < 0.001). **(B)** The cophenetic, RSS and dispersion distributions with rank = 2–10; combining these indicators results in the optimal number of clusters of 2. **(C)** Consensus map of NMF clustering. **(D)** Survival analyses for the two adipose subtypes based on 530 patients with ccRCC from TCGA cohorts including 166 cases in cluster A, and 364 cases in cluster B Kaplan-Meier curves with log-rank *p*  = 0.003 showed a significant survival difference among two adipose clusters. The cluster A showed significantly worse overall survival than the cluster B. **(E)** NMF clustering of 49 ARGs in the TCGA ccRCC cohort. The adipose subtypes, TNM stages, clinical stages, survival status and age were used as patient annotations. Red represented high expression of ARGs and blue represented low expression. **(F)** Proportion of cases with different stages (chi-square test, *p* = 0.006) and difference of RARRES2 mRNA expression (wilcoxon test, *p* < 0.001) in the two adipose subtypes.

A total of 49 ARGs were finally screened for differential expression between normal and ccRCC tissues. Compared to normal kidney tissues, 33 ARGs demonstrated markedly higher expression in ccRCC tissues (e.g., APOC1 and ENO2), while the rest were on the contrary (e.g., SFRP1 and DAG1) (**Figure 2A**). The above analyses indicated that the expression imbalance of ARGs played a crucial role in the occurrence, progression and adipogenic transdifferentiation of ccRCC. According to the cophenetic, dispersion and silhouette indicators, two clusters were eventually identified using NMF clustering (**Figures 2B, C**), including 169 cases in cluster A and 370 cases in cluster B. Then, we integrated the clinical information and found that patients in cluster A showed a significantly worse OS than cluster B (log-rank *p* = 0.003; **Figures 2D**). **Figure 2E** showed a heatmap displaying the clinic-pathological features and expression distributions of differentially expressed ARGs. Furthermore, we explored the proportion of different levels of pathological stages in two subtypes, as well as the expression of RARRES2. And it turned out that patients in cluster A exhibited significantly more advanced clinical stages (*p* = 0.009) and a markedly higher RARRES2 expression (*p* < 0.001) than those in cluster B (**Figure 2F**).

### Biological and Immune-Related Features in Adipose Subtypes

**Figure 3:**
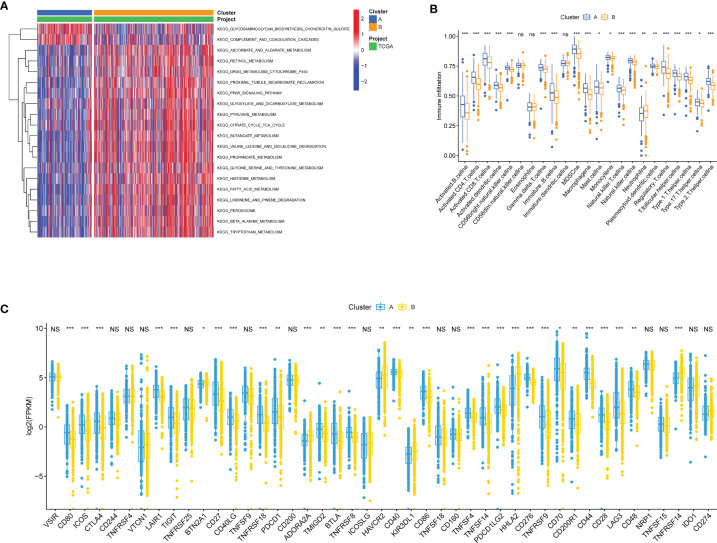
Biological and immune-related characteristics in different clusters. **(A)** GSVA showing the activation states of biological pathways in two clusters. The heatmap was used to visualize these biological processes, and red represented activated pathways and blue represented inhibited pathways. **(B)** The abundance of each TME infiltrating cell in cases in two clusters. The upper and lower ends of the boxes represented interquartile range of values. The lines in the boxes represented median value, and colorful dots showed outliers. The asterisks represented the statistical *p* value (^*^ *p* < 0.05, ^**^ *p* < 0.01, ^***^ *p* < 0.001; ns, Not Statistically Significant). **(C)** The expression of ICP mRNAs in two clusters. The upper and lower ends of the boxes represented interquartile range of values. The lines in the boxes represented median value. The asterisks represented the statistical *p* value (^*^ *p* < 0.05, ^**^ *p* < 0.01, ^***^ *p* < 0.001; NS, Not Statistically Significant).

To investigate the molecular differences in participating KEGG pathways between the two subtypes, we utilized GSVA to analyze the enriched pathways in each cluster. Some of the results were shown in **Figure 3A** (**Table S1** for more details). Cluster A was enriched with less nutrient metabolism pathways, for instance, metabolisms of pyruvate (adjusted *p* value = 2.05E-15), glycine, serine and threonine (adjusted *p* value = 1.67E-12), and fatty acid (adjusted *p* value = 9.99E-26). In addition, we noticed that PPAR signaling pathway also presented less in cluster A (adjusted *p* value = 3.95E-16), which was involved biological processes such as lipid metabolism gene expression, lipid oxidation, and promotion of adipocyte differentiation. ssGSEA was performed to analyze the proportion of 23 kinds of immune cells in immune infiltration microenvironment between adipose subtypes. As was shown in **Figure 3B**, all immune cells have some degree of difference between samples in cluster A and B, except the CD56dim natural killer cell, Eosinophil, and Immature dendritic cell. ccRCC patients in cluster A had higher scores in 19 kinds of immune cells, among which Myeloid-derived suppressor cells (MDSC), Monocyte and Activated CD8 T cell appeared to be the top three cell types. Previous studies have shown that immune checkpoints (ICPs) played a vital role in tumor immunity. Therefore, we explored the expression of ICPs in two clusters. Compared to samples in cluster B, 25 ICP genes demonstrated markedly higher expression in cluster A (e.g., CTLA4 and PDCD1), while 6 expressed significantly less (e.g., BTN2A1 and ADORA2A) (**Figure 3C**). These results indicated that immune surveillance of T cells was repressed in cluster A patients, thus promoting the growth of tumor.

### Construction of ADI Model Based on ARGs

**Figure 4:**
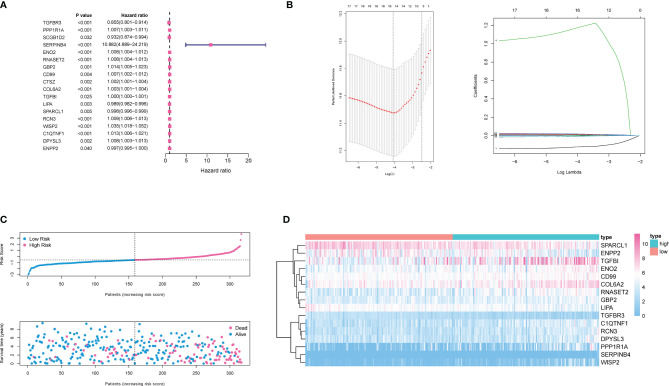
Construction of ADI model. **(A)** 18 genes associated with OS of ccRCC patients were obtained through univariate Cox regression analysis. **(B)** A 16-mRNA signature was constructed by LASSO Cox regression. **(C)** Prognostic analysis of 16-gene signature in the training set. The dotted line represented the median risk score and divided the patients into low- and high-risk group. More dead patients corresponded to the higher risk score according to the curve of risk score and survival status of the patients. **(D)** Heatmap of the expression profiles of the 16 prognostic genes in low- and high-risk group.

For these differentially expressed ARGs and survival data, a univariate Cox regression was performed, and 18 genes with significant prognostic differences were identified (**Figure 4A**). We then utilized a Lasso-Cox regression model to further select the ARGs with the highest prognostic value. (**Figure 4B**) Finally, a prognosis signature based on the personalized expression level of 16 ARGs was constructed. Using the coefficients obtained from the Lasso Cox regression model, we defined an index–ADI to calculate the risk score of each patient. The formula was as follows:


$$\begin{array}{c} ADI=(-0.124031)\times \exp^{TGFBR3}+0.001859\times \exp^{PPP1R1A} \end{array}$$

Based on the median ADI, ccRCC patients with ADIs higher than the median were defined as a high-risk group, and ADIs lower than the median were defined as a low-risk group. The ADI distribution, survival time and survival status, and expression of 16 genes in the training cohort were shown in **Figures 4C** and **D**. Samples with high ADIs had significantly lower OS than those with low ADIs, indicating that the patients with high ADIs had a worse prognosis. High expression of 12 ARGs (PPP1R1A, SERPINB4, ENO2, RNASET2, GBP2, COL6A2, COL6A2, TGFBI, RCN3, WISP2, C1QTNF1, DPYSL3, and ENPP2) was associated with high risk, consistent with their positive coefficients in our formula, so these genes were considered risk factors.

### Validation of the Robustness of the Model in Both Internal and External Cohorts

**Figure 5:**
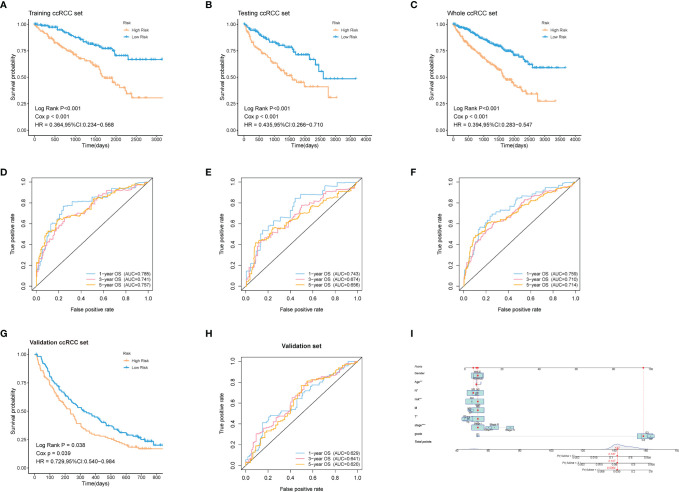
Validation of the robustness of the model in both internal and external cohorts. **(A–C, G)** Kaplan–Meier survival analysis of the training ccRCC set, testing ccRCC set, whole ccRCC set, and validation set (NIHMS1611472 from NCBI). The survival rate of the patients in the high-risk group was significantly lower than those in the low-risk group respectively. **(D–F, H)** Time-dependent ROC analysis of the training ccRCC set, testing ccRCC set, whole ccRCC set, and validation set (NIHMS1611472 from NCBI). The AUC suggested that the prognostic accuracy of the 16-mRNA signatures in the discovery set was robust and accurate. **(I)** The nomogram for predicting proportion of patients with 3- or 5-year OS. The asterisks represented the statistical p value (*p < 0.05, **p < 0.01, ***p < 0.001).

60% of the 530 TCGA preprocessed samples were randomly selected as the training cohort to construct the above model. As was revealed in the Kaplan-Meier (KM) curve, patients in the high-risk group had a worse prognosis compared to those in the low-risk group in the training cohort (log-rank *p* < 0.001) (**Figure 5A**). The rest 40% samples were taken as the testing cohort. We applied the same model and coefficients as the training cohort to generate the ADI of each patient in both the testing cohort and the whole cohort from TCGA. Consistent with the results from the training set, significantly higher survival rates were observed in low-risk groups in comparison with the high-risk ones, both in the testing cohort (log-rank *p* < 0.001; **Figure 5B**), and the whole cohort (log-rank *p* < 0.001; **Figure 5C**). The survival time-dependent receiver operating characteristic (ROC) curves in the training, testing, and whole cohorts were also performed. **Figures 5D–F** exhibited the prognostic classification efficiency in three cohorts at 1-year, 3-years, and 5-years respectively. The areas under the curve (AUC) of the 1 year, 3 years, and 5 years ROC curve in the training cohort were 0.785, 0.741, and 0.757, with 0.743, 0.674, and 0.656 in the testing cohort and 0.759, 0.710, and 0.714 in the whole cohort. Using data from the NIHMS1611472 dataset, we also drew KM survival curve distribution of 16 ARGs signature (**Figure 5G**) and performed the ROC analysis of the prognostic classification of the risk score (**Figure 5H**). The AUC for 1-year, 3-years, and 5-years were 0.629, 0.641, and 0.620. There was a significant difference between the high- and low-risk groups (log-rank *p* = 0.038). The results of validation of the robustness of our model in both internal and external cohorts advocated that our risk model was accurate in the forecasting of survival. In addition, a nomogram including our risk signature and some clinic-pathological factors was established to improve clinical feasibility (**Figure 5I**).

### Survival Analysis in Stratification of Different Clinical Features

**Figure 6:**
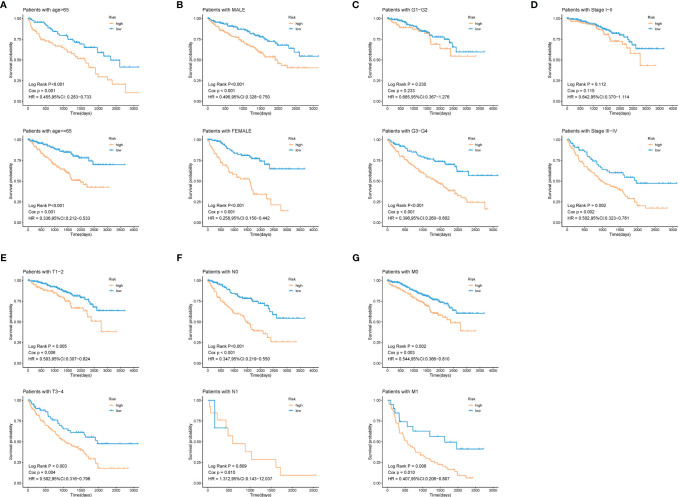
Validation of the prognostic efficacy of our model under the stratifications of different clinical parameters. **(A)** age > 65 and age <=65, **(B)** male and female, **(C)** histological grade 1/2 and 3/4, **(D)** clinical stage I/II and III/IV, **(E)** T 1/2 and 3/4, **(F)** N 0 and 1, **(G)** M 0 and 1.

To explore whether patients at different risks presented a distinct prognosis, patients were further stratified into subgroups of age ≤ 65 and age> 65 years old, male and female, histological grade I/II and grade III/IV, stage I/II and stage III/IV, T1/2 and T3/4, N0 and N1, M0 and M1. As was shown in **Figure 6**, except for the subgroup of histological grade I/II (log-rank *p* = 0.23), clinical stage I/II (log-rank *p* = 0.112), and N1 (log-rank *p* = 0.809), patients in high-risk group showed a worse prognosis than those in low-risk groups. This further indicated that our model still had a strong predictive ability in different clinical signs.

### Immune-Related Feature and Prognostic Value of the Key Modeling Gene

**Figure 7:**
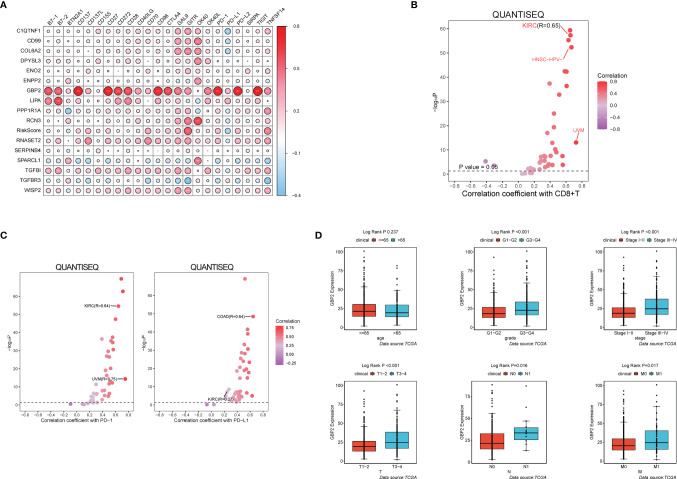
GBP2 was associated with the tumor microenvironment of ccRCC. **(A)** The correlation matrix of each immune checkpoint in modeling genes. (^*^ *p* < 0.05, ^**^ *p* < 0.01, ^***^ *p* < 0.001). QuanTIseq analysis of RNA-seq data from 19 TCGA solid cancers: **(B)** Correlation of GBP2 expression with CD8^+^ T cells, **(C)** Correlation of GBP2 expression with immune checkpoint PD-1 and PD-L1. **(D)** Higher GBP2 expression was associated with more severe clinical parameters in ccRCC patients such as histological grade, clinical stage, T classification., lymph nodes, distant metastasis according to data from TCGA.

**Figure 8:**
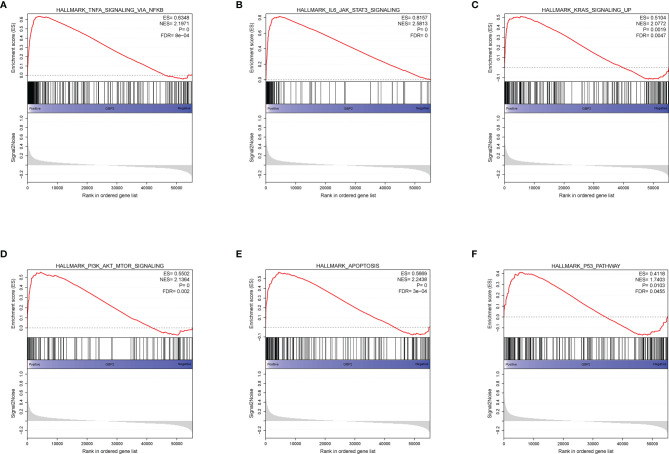
GSEA analysis revealed several activated oncogenic pathways associated with the expression of GBP2. **(A)** TNF-α signaling *via* NF-κB. **(B)** IL-6-JAK-STAT3 signaling. **(C)** KRAS signaling. **(D)** PI3K/AKT/mTOR signaling. **(E)** Apoptosis. **(F)** P53 pathway.

During the growth and progression of tumors, they often exhibited immunosuppression. Considered as the main mechanism of tumor immune resistance, the immune checkpoint pathways allowed tumors to negatively regulate T cells to escape immune surveillance. As a promising immunotherapy, the immune checkpoint blockade has been applied to various tumors including ccRCC. Therefore, we investigated the correlation between expressions of ICPs and risk scores and expressions of our modeling genes by using Pearson correlation analysis in TCGA cohort (**Figure 7A**). And we noticed that one gene GBP2 was correlated positively with the expressions of all listed immune checkpoint except CD155 or OX40. Speculating that GBP2 might play a role in the tumor microenvironment, we analyzed its correlation with the CD8^+^ T cells, the most potent effector cell in immunotherapy. As was shown in **Figure 7B**, GBP2 and CD8^+^ T cell represented good correlation in ccRCC samples (R = 0.65, *p* < 0.05). Similarly, the significant correlation coefficients of GBP2 expression with classical immune checkpoints PD-1 and PD-L1 were presented in **Figure 7C**. We also evaluated the relationship between GBP2 expression and clinical characteristic subtypes in TCGA ccRCC samples. Other than age, the expression of GBP2 was significantly higher in subgroups with more severe clinical predictors, including histological grade (log-rank *p* < 0.001), clinical stage (log-rank *p* < 0.001), T classification (log-rank *p* < 0.001), lymph nodes (log-rank *p* = 0.016), and distant metastasis (log-rank *p* = 0.017) (**Figure 7D**). GSEA was utilized to biological changes associated with the expression of GBP2. **Figures 8A–F** revealed the markedly different signaling pathways, including the TNFα signaling *via* NF-κB, IL6-JAK-STAT3 signaling, KRAS signaling, PI3K-AKT-mTOR signaling, apoptosis, and p53 pathway. These were primarily correlated with carcinogenesis, invasion, and the immune microenvironment of tumor cells.

### Validation of the Role of GBP2 at the Translational Level and Through Experiments

**Figure 9:**
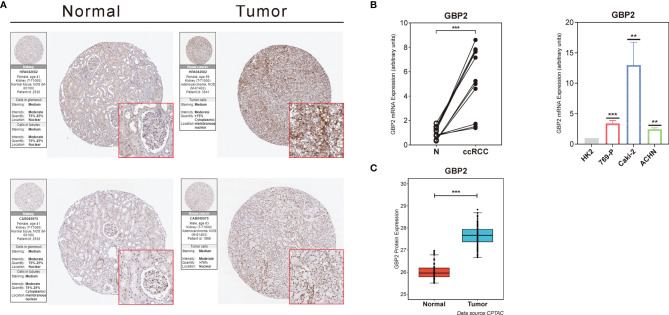
Validation of the role of GBP2 at the translational and transcriptional levels. **(A)** Immunohistochemical images from the HPA database show GBP2 protein expression in normal kidney (Normal) and KIRC (Tumor) tissues by different antibodies. **(B)** The mRNA expression of GBP2 was significantly different between normal kidney and ccRCC tissues according to the PCR results, as well as non-renal cancer cell line and several RCC cell lines. **(C)** The protein GBP2 expression of GBP2 was significantly different between normal kidney and ccRCC tissues according to the data from CPTAC. The asterisks represented the statistical p value (**p < 0.01, ***p < 0.001).

The HPA database was used to confirm the protein expression between ccRCC and normal tissues. The protein expression of GBP2 was higher in the tumor tissues compared to the normal tissue, which was consistent with our results from RT-qPCR (**Figures 9A, B**). In addition, the expression of GBP2 was always significantly elevated in kidney cancer cell lines (769-P, Caki-2, and ACHN) compared to the normal human renal tubular epithelial HK-2 cells (**Figure 9B**). It was insufficient to use genomic data to predict cancer prognosis while proteomics could improve our understanding of the etiology and progression of cancer and improve the assessment of cancer prognosis. The data from CPTAC showed that there were significant differences in the translational level of GBP2 between normal tissues and ccRCC (**Figure 9C**).

## Discussion

Traditional prognostic factors for ccRCC mainly included tumor-node-metastasis (TNM) stage, histological grade, and clinical stage. Although these indicators have been progressively refined over the past decades, there are still many difficulties in reducing the mortality and improving prognosis of patients with ccRCC. The identification of novel diagnostic and prognostic biomarkers for ccRCC is a necessity at present.

Dysregulated metabolism was a hallmark of malignant tumor, manifested through alterations in metabolites. Based on metabolomic profiling, Hakimi et al. found that ccRCC characterized broad shifts of central carbon metabolism, one-carbon metabolism and anti-oxidant response (15). Many prognostic models were developed according to the important alterations in metabolic processes in ccRCC. Gui et al. (16) developed a hypoxia-immune–based multiomics signature since they noticed both the hypoxia and immune status of the tumor microenvironment in ccRCC. A predictive model consisting of 13 glycolysis-related genes was also constructed by Zhang et al. (17) based on the high levels of glycolysis in tumor cells even under aerobic conditions. Moreover, recent studies shed light on the possible mechanisms by which deregulated lipid metabolism promotes malignant proliferation and adipogenic transdifferentiation in ccRCC (7, 18, 19), while the relationship between the degree of adipogenic transdifferentiation of tumor cells and the disease prognosis of ccRCC still remained unclear. These results brought us a novel research direction.

In the current study, we first verified that the distribution of adipokine chemerin promoting cellular adiposity differentiation differed significantly between clinical subgroups, and that the gene RARRES2 was expressed more in the advanced subgroups. This demonstrated a positive correlation between the degree of tumor cell adipogenic transdifferentiation and the severity of the disease, which was consistent with the previous findings (8). Next, based on extracted 49 differentially expressed genes from genes encoding specific proteins secreted by adipocytes, we defined two subtypes with distinct clinical and biological features in ccRCC. The results showed that patients in cluster A had worse clinical outcomes and a greater proportion of advanced stages. Moreover, cluster A exhibited a significant lipid metabolism inactivation status, including the poorly expressed fatty acid metabolism and PPAR signaling pathways, which played a role in clearing cellular lipids in kidney tissues. Previous studies have demonstrated that the infiltrating immune cells were essential for tumor growth, metastasis, and drug resistance (20, 21). When we performed immune cell infiltration analysis of the tumor microenvironment for both clusters, we found that cluster A was enriched in both innate and acquired immune cell infiltration, while patients in cluster A didn’t show a matching survival advantage. Given that samples in cluster A expressed significantly more immune checkpoints, we hypothesized that immunotherapy might have good efficacy for ccRCC patients with high adipogenic transdifferentiation and severe condition.

Based on the results of univariate Cox regression analysis, 18 ARGs associated with OS were picked out. To eliminate the limitation of overfitting and further optimize gene selection, Lasso regression analysis was performed and finally 16 independent prognostic ARGs (TGFBR3, PPP1R1A, SERPINB4, ENO2, RNASET2, GBP2, CD99, COL6A2, TGFBI, LIPA, SPARCL1, RCN3, WISP2, C1QTNF1, DPYSL3 and ENPP2) were screened out to develop the ADI. After constructing the 16-gene signature, patients were separated into high-risk and low-risk groups. Our results showed that high risk-score patients had an inferior survival probability and clinical outcomes. Not only that, but we also verified that our model had a strong predictive efficacy through the validation of its robustness in both internal and external cohorts.

The guanylate-binding proteins (GBPs) were a large subfamily within the dynamin superfamily of large guanosine triphosphatase (GTPases) and involved in the regulation of intracellular immunity and basic physiological processes (22). As a member of the GBPs, GBP2 was important for protective immunity against microorganisms and viral pathogens (23). Our results showed that the expression GBP2 was significantly and positively correlated with both the expression of most immune checkpoints (such as PD-1 and PD-L1) and the distribution of CD8^+^ T cells in the RCC tumor microenvironment. In addition, stratified analysis revealed that ccRCC patients with significantly upregulated expression of GBP2 tended to be at a more advanced stage. These findings suggested that GBP2 might be a potential biomarker for ccRCC disease assessment and immunotherapy response prediction. To investigate the molecular mechanisms underlying the regulation of GBP2 in ccRCC, we performed GSEA analysis to identify the GBP2-related enriched biological processes and pathways. The GSEA results suggested that TNFα signaling *via* NF-κB, IL6-JAK-STAT3 signaling, KRAS signaling, PI3K-AKT-mTOR signaling, correlated with progression of ccRCC. It has been showed that there was a direct correlation between tumor grade, invasion and metastasis of RCC and the expression and activation of NF-κB (24). Su et al. have found that insulin-like growth factor I (IGF-I) exerted stimulative role in RCC cell growth and had suppressive effects on RCC cell apoptosis through JAK2/STAT3 pathway (25). While mutation of KRAS was a rare event in RCC, wild type KRAS could exert tumor suppressor effects on RCC cell proliferation and tumor growth (26). Overactivation of PI3K-Akt-mTOR signaling has been suggested to correlate with aggressive behavior and poor prognosis in RCC tumors (27). All these results suggested that GBP2 promoted oncogenesis and progression of ccRCC through regulating multiple signaling pathways and it was validated at both the transcriptional and translational level.

Recent studies have explored the role of GBP2 on carcinogenesis and found that GBP2 could enhance the invasive ability of glioblastoma *via* the GBP2/Stat3/FN1 signal cascade (28). It was reported that the overexpression of GBP2 was correlated with an advanced T classification and poor OS in pancreatic adenocarcinoma (29). However, when it comes to colorectal cancer, GBP2 was proven to inhibit the growth of cancer cells by interfering with Wnt signal transduction. Godoy et al. found that the high expression of GBP2 was associated with a better prognosis in breast cancer (30). This might be due to the fact that GBP2 could prevent dynamin-related protein 1 (Drp1) translocating from the cytoplasm to the mitochondria, thus weakening the Drp1-dependent mitochondrial fission and the invasion of breast cancer cells (31). Additionally, in the present study, we found that GBP2 was a promising biomarker for the prediction of ccRCC prognosis and response to immunotherapy.

The association between excess body weight and risk of renal cell carcinoma has been widely reported in large prospective cohorts (32, 33). Tan and his colleagues found that chemerin could promote the progression of ccRCC by inducing the adipogenic transdifferentiation of tumor cells. Meanwhile, the level of chemerin in the peripheral circulation was positively correlated with the body mass index (BMI) of patients, confirming a positive relationship between obesity and adipogenic transdifferentiation of ccRCC (8). However, the BMI cannot provide any information about the distribution of adipose tissues. Federico et al. explored the relationship between the distribution of adipose tissues and subtypes of RCC, and confirmed that ccRCC patients tended to exhibit a greater amount of abdominal fat, especially visceral adipose tissues (VATs) (34). The current theory suggests that adipose tissue is an endocrine/metabolic organ (35). Indeed, VATs possess higher hormonal and metabolic functions compared to subcutaneous adipose tissues (SATs) and can secrete a variety of factors conducive to the adipogenic transdifferentiation of surrounding cells (8, 34). Therefore, visceral obesity, especially perirenal fat, may serve as a positive role in promoting the adipogenic transdifferentiation of ccRCC (36, 37). In essence, kidney cancer cells and perirenal adipose tissue can interact through genomic changes and regulation (8, 38). In addition, Greco’s study found a different distribution of perirenal adipose tissues between ccRCC and non-ccRCC. Specifically, ccRCC had more abundant perirenal fat, which might partly explain the mild degree of adipogenic transdifferentiation and the low expression level of chemerin in non-ccRCC cases, where perirenal adipose tissues were less distributed (8, 39).

The advantages of our study lied in our statistical analysis of adipokine-related genetic prognostic signature using high-throughput data and large-scale databases, which catered to the urgent need for validated index of ccRCC. In addition, our study could contribute to a better understanding of the role of adipogenic differentiation in ccRCC. Inevitably, our study also has several limitations. Firstly, clinical parameters such as age, pathological stage were not integrated into our ADI formula. Secondly, the clinical data in TCGA database was not comprehensive, and we were unable to obtain more parameters to validate our model, such as CT images and nephrometry scores. Thirdly, the mechanism of ARGs such as GBP2 affecting the occurrence and development of ccRCC needed further study *in vivo* and in *vito*.

## Conclusion

In conclusion, the adipogenic transdifferentiation status of tumor cells in patients with ccRCC was closely related to prognosis. We have presented a comprehensive analysis for ARG expression profiles and clinical data and identified a 16-mRNA signature that could effectively predict the prognosis of patients in ccRCC patients. ARGs such as GBP2 could helpfully provide insights into the underlying mechanism of ccRCC and may be a novel independent biomarker in the prediction of the survival of ccRCC patients.

## Data Availability Statement

The original contributions presented in the study are included in the article/**Supplementary Material**. Further inquiries can be directed to the corresponding author.

## Ethics Statement

The studies involving human participants were reviewed and approved by The Ethical Committee of The First Affiliated Hospital of Nanjing Medical University. The patients/participants provided their written informed consent to participate in this study.

## Author Contributions

NS designed this work. SW and XW wrote the manuscript. CJ performed the bioinformatics analysis. YW, XZ, and RC performed the data review. All authors have read and approved the manuscript.

## Funding

This work was supported by the National Natural Science Foundation of China (grant number 82071638).

## Conflict of Interest

The authors declare that the research was conducted in the absence of any commercial or financial relationships that could be construed as a potential conflict of interest.

## Publisher’s Note

All claims expressed in this article are solely those of the authors and do not necessarily represent those of their affiliated organizations, or those of the publisher, the editors and the reviewers. Any product that may be evaluated in this article, or claim that may be made by its manufacturer, is not guaranteed or endorsed by the publisher.
