# Supplementary material for: Adipogenic Transdifferentiation and Regulatory Factors Promote the Progression and the Immunotherapy Response of Renal Cell Carcinoma: Insights From Integrative Analysis
Source: Front Oncol. 2022 Mar 9;12:781932. doi: 10.3389/fonc.2022.781932 (PMC8959453; doi:10.3389/fonc.2022.781932)
Supplement: Supplementary file 1 [file DataSheet_1.pdf]

Table S1: Signaling pathways with significant differences between the two clusters

id	logFC	AveExpr	t	P.Value	adj.P.Val	B
KEGG_FATTY_ACID_METABOLISM	0.432339	-0.02108	12.41621	2.55E-31	4.74E-29	60.31862
KEGG_PROXIMAL_TUBULE_BICARBONATE_RECLAMATION	0.339627	-0.03177	11.53722	1.11E-27	1.03E-25	52.06801
KEGG_LIMONENE_AND_PINENE_DEGRADATION	0.47803	-0.02534	11.30899	9.18E-27	5.69E-25	49.98452
KEGG_HISTIDINE_METABOLISM	0.306527	-0.03269	10.93475	2.79E-25	1.30E-23	46.62399
KEGG_VALINE_LEUCINE_AND_ISOLEUCINE_DEGRADATION	0.431296	-0.02036	10.72071	1.90E-24	7.08E-23	44.73431
KEGG_BUTANOATE_METABOLISM	0.35041	-0.01945	10.53451	9.92E-24	3.08E-22	43.11005
KEGG_PROPANOATE_METABOLISM	0.40359	-0.02501	10.47602	1.66E-23	4.41E-22	42.60362
KEGG_PPAR_SIGNALING_PATHWAY	0.254104	-0.02053	10.36449	4.41E-23	1.03E-21	41.64322
KEGG_BETA_ALANINE_METABOLISM	0.351551	-0.02491	10.2003	1.83E-22	3.79E-21	40.24183
KEGG_ASCORBATE_AND_ALDARATE_METABOLISM	0.297955	0.01858	9.93782	1.73E-21	3.23E-20	38.03312
KEGG_TRYPTOPHAN_METABOLISM	0.293304	-0.02818	9.850494	3.63E-21	6.14E-20	37.30705
KEGG_RETINOL_METABOLISM	0.196517	0.019647	8.690664	4.30E-17	6.67E-16	28.10156
KEGG_PYRUVATE_METABOLISM	0.276161	-0.03749	8.591659	9.21E-17	1.32E-15	27.35525
KEGG_GLYCINE_SERINE_AND_THREONINE_METABOLISM	0.246997	-0.03471	8.082195	4.18E-15	5.40E-14	23.61874
KEGG_PEROXISOME	0.281616	-0.02544	8.076661	4.36E-15	5.40E-14	23.57913
KEGG_GLYCOSAMINOGLYCAN_BIOSYNTHESIS_CHONDROITIN_SULFATE	-0.26509	-0.03311	-7.86104	2.07E-14	2.41E-13	22.05229
KEGG_DRUG_METABOLISM_CYTOCHROME_P450	0.172566	0.001178	7.535893	2.05E-13	2.25E-12	19.81219
KEGG_COMPLEMENT_AND_COAGULATION_CASCADES	-0.20224	-0.00507	-7.49666	2.70E-13	2.79E-12	19.54707
KEGG_GLYOXYLATE_AND_DICARBOXYLATE_METABOLISM	0.236529	-0.04009	7.112213	3.64E-12	3.56E-11	17.0088
KEGG_CITRATE_CYCLE_TCA_CYCLE	0.314398	-0.02075	7.095638	4.06E-12	3.77E-11	16.90184
KEGG_TASTE_TRANSDUCTION	-0.12463	-0.01755	-7.02668	6.39E-12	5.66E-11	16.45909
KEGG_METABOLISM_OF_XENOBIOTICS_BY_CYTOCHROME_P450	0.165014	0.001231	7.010513	7.11E-12	6.01E-11	16.35578
KEGG_ONE_CARBON_POOL_BY_FOLATE	0.239589	-0.03342	6.943827	1.10E-11	8.88E-11	15.93184
KEGG_TERPENOID_BACKBONE_BIOSYNTHESIS	0.252042	-0.03105	6.835492	2.21E-11	1.71E-10	15.25035
KEGG_ALANINE_ASPARTATE_AND_GLUTAMATE_METABOLISM	0.20612	-0.03576	6.683606	5.81E-11	4.33E-10	14.31007
KEGG_GLYCOLYSIS_GLUCONEOGENESIS	0.204412	-0.02556	6.454196	2.42E-10	1.73E-09	12.92389
KEGG_P53_SIGNALING_PATHWAY	-0.17863	-0.0311	-6.3815	3.77E-10	2.60E-09	12.49327
KEGG_GLYCEROLIPID_METABOLISM	0.14895	-0.03453	6.356726	4.38E-10	2.91E-09	12.34748
KEGG_ARGININE_AND_PROLINE_METABOLISM	0.180967	-0.02587	6.294712	6.37E-10	4.09E-09	11.98467
KEGG_ECM_RECEPTOR_INTERACTION	-0.19719	-0.02269	-6.23013	9.38E-10	5.81E-09	11.61009
KEGG_FOLATE_BIOSYNTHESIS	0.210706	-0.02794	6.150549	1.50E-09	9.01E-09	11.15313
KEGG_CYTOKINE_CYTOKINE_RECEPTOR_INTERACTION	-0.14246	-0.02514	-6.0819	2.25E-09	1.31E-08	10.76306
KEGG_PENTOSE_AND_GLUCURONATE_INTERCONVERSIONS	0.171837	0.00795	5.948403	4.87E-09	2.74E-08	10.01539
KEGG_NITROGEN_METABOLISM	0.139064	-0.01486	5.891014	6.75E-09	3.69E-08	9.698448
KEGG_BASAL_CELL_CARCINOMA	-0.13972	-0.03349	-5.85002	8.52E-09	4.53E-08	9.473726
KEGG_GLYCOSAMINOGLYCAN_BIOSYNTHESIS_KERATAN_SULFATE	-0.20378	-0.0253	-5.7729	1.31E-08	6.79E-08	9.054633
KEGG_HYPERTROPHIC_CARDIOMYOPATHY_HCM	-0.14864	-0.02569	-5.74232	1.56E-08	7.83E-08	8.889875

KEGG_STEROID_HORMONE_BIOSYNTHESIS	0.120141	0.00901	5.73761	1.60E-08	7.83E-08	8.864536
KEGG_RENIN_ANGIOTENSIN_SYSTEM	0.180471	-0.02278	5.661731	2.43E-08	1.16E-07	8.459248
KEGG_DILATED_CARDIOMYOPATHY	-0.15257	-0.025	-5.64861	2.62E-08	1.22E-07	8.389676
KEGG_BIOSYNTHESIS_OF_UNSATURATED_FATTY_ACIDS	0.174101	-0.03986	5.453365	7.53E-08	3.42E-07	7.371013
KEGG_LYSINE_DEGRADATION	0.196624	-0.02445	5.430998	8.48E-08	3.76E-07	7.256364
KEGG_TYROSINE_METABOLISM	0.119048	-0.03316	5.410516	9.45E-08	4.09E-07	7.15175
KEGG_REGULATION_OF_AUTOPHAGY	0.132946	-0.00386	5.372428	1.16E-07	4.89E-07	6.958145
KEGG_PRION_DISEASES	-0.15653	-0.01514	-5.26952	1.98E-07	8.18E-07	6.441214
KEGG_PARKINSONS_DISEASE	0.180866	-0.03866	5.250817	2.18E-07	8.81E-07	6.348232
KEGG_OXIDATIVE_PHOSPHORYLATION	0.193528	-0.03772	5.239984	2.31E-07	9.12E-07	6.294508
KEGG_PRIMARY_IMMUNODEFICIENCY	-0.19116	-0.03151	-5.10618	4.56E-07	1.77E-06	5.639284
KEGG_HEMATOPOIETIC_CELL_LINEAGE	-0.14661	-0.01576	-5.02362	6.90E-07	2.62E-06	5.242619
KEGG_INTESTINAL_IMMUNE_NETWORK_FOR_IGA_PRODUCTION	-0.17924	-0.02797	-4.9885	8.21E-07	3.06E-06	5.075713
KEGG_GLYCOSPHINGOLIPID_BIOSYNTHESIS_GLOBO_SERIES	-0.15642	-0.02192	-4.83851	1.71E-06	6.23E-06	4.374798
KEGG_SYSTEMIC_LUPUS_ERYTHEMATOSUS	-0.12408	-0.0205	-4.82894	1.79E-06	6.40E-06	4.330735
KEGG_PANTOTHENATE_AND_COA_BIOSYNTHESIS	0.153582	-0.02711	4.764103	2.44E-06	8.56E-06	4.034342
KEGG_PRIMARY_BILE_ACID_BIOSYNTHESIS	0.136156	-0.00719	4.720436	3.00E-06	1.03E-05	3.836812
KEGG_ADIPOCYTOKINE_SIGNALING_PATHWAY	0.133907	-0.02655	4.551566	6.58E-06	2.21E-05	3.0887
KEGG_HUNTINGTONS_DISEASE	0.13764	-0.03529	4.54884	6.66E-06	2.21E-05	3.076833
KEGG_HEDGEHOG_SIGNALING_PATHWAY	-0.10304	-0.02912	-4.52543	7.42E-06	2.42E-05	2.975162
KEGG_ARRHYTHMOGENIC_RIGHT_VENTRICULAR_CARDIOMYOPATHY_ARVC	-0.13102	-0.02551	-4.44536	1.07E-05	3.42E-05	2.631137
KEGG_PHENYLALANINE_METABOLISM	0.121937	-0.03335	4.286922	2.15E-05	6.65E-05	1.967191
KEGG_HOMOLOGOUS_RECOMBINATION	-0.13268	-0.04292	-4.03292	6.30E-05	0.000192	0.949857
KEGG_LEISHMANIA_INFECTION	-0.14611	-0.02247	-4.01959	6.66E-05	0.0002	0.898102
KEGG_FRUCTOSE_AND_MANNOSE_METABOLISM	0.129195	-0.02749	3.968289	8.22E-05	0.000243	0.700333
KEGG_PORPHYRIN_AND_CHLOROPHYLL_METABOLISM	0.118721	-0.00423	3.932782	9.49E-05	0.000276	0.564861
KEGG_PATHOGENIC_ESCHERICHIA_COLI_INFECTION	-0.13015	-0.02185	-3.84977	0.000132	0.000379	0.252629
KEGG_CELL_CYCLE	-0.12283	-0.02989	-3.67186	0.000265	0.000746	-0.39526
KEGG_VIRAL_MYOCARDITIS	-0.11482	-0.02734	-3.60573	0.00034	0.000944	-0.62869
KEGG_ASTHMA	-0.12848	-0.02696	-3.45263	0.000599	0.001513	-1.15349
KEGG_SELENOAMINO_ACID_METABOLISM	0.103519	-0.03047	3.216846	0.001374	0.003318	-1.91908
KEGG_DNA_REPLICATION	-0.12892	-0.03223	-3.21217	0.001396	0.003328	-1.93375
KEGG_FC_GAMMA_R_MEDIATED_PHAGOCYTOSIS	-0.10245	-0.02025	-3.15176	0.001713	0.004033	-2.12126
KEGG_CIRCADIAN_RHYTHM_MAMMAL	0.113661	-0.03463	3.09525	0.002068	0.004749	-2.29354
KEGG_GLYCOSPHINGOLIPID_BIOSYNTHESIS_GANGLIO_SERIES	-0.10819	-0.00998	-3.08123	0.002166	0.004914	-2.33583
KEGG_VASOPRESSIN_REGULATED_WATER_REABSORPTION	0.111638	-0.01996	3.016728	0.002675	0.005854	-2.52796
KEGG_RIBOSOME	-0.15173	-0.01604	-2.97457	0.003065	0.006629	-2.65141
KEGG_VALINE_LEUCINE_AND_ISOLEUCINE_BIOSYNTHESIS	0.119756	-0.025	2.95411	0.003272	0.006916	-2.71072
KEGG_TYPE_I_DIABETES_MELLITUS	-0.11879	-0.02429	-2.94023	0.00342	0.00705	-2.75072

KEGG_ALLOGRAFT_REJECTION	-0.1042	-0.03115	-2.34404	0.019437	0.034107	-4.29596
